# Global Trends in Heart Failure Self-Care Research: A Bibliometric and Visual Analysis

**DOI:** 10.3390/healthcare14172881

**Published:** 2026-09-07

**Authors:** Xiaoyue Wei, Xiu Jin, Zeya Wu, Rong She, Guotao Sun, Yan Sun, Pei Yang, Huaping Wei

**Affiliations:** 1 School of Nursing, Lanzhou University, Lanzhou 730000, China; 2 Ward 1, Cardiovascular Medicine, The First Hospital of Lanzhou University, Lanzhou 730000, China; 3 School of Nursing, Gansu University of Chinese Medicine, Lanzhou 730000, China; 4 Intensive Care Unit, The First Affiliated Hospital of Army Medical University, Chongqing 400030, China; 5 Outpatient Department, The First Hospital of Lanzhou University, Lanzhou 730000, China

**Keywords:** heart failure, self-care, bibliometric analysis, CiteSpace, VOSviewer

## Abstract

**Background:** Adequate patient self-care is fundamental to effective heart failure (HF) management and is endorsed by international guidelines to improve clinical outcomes. Despite a growing body of evidence, the global research landscape, including publication trends, collaborative networks, and research hotspots, remains poorly characterized. **Methods:** Publications on HF self-care were extracted from the Web of Science Core Collection from database inception to 29 June 2026, restricted to the categories of “Nursing” or “Cardiac & Cardiovascular Systems” and to English-language articles and reviews. Bibliometric analyses were performed using VOSviewer and CiteSpace for specific characteristics such as publication year, countries, institutions, authors, journals, keywords, and co-cited references. **Results:** A total of 2867 publications were included, revealing a sustained increase in annual output and citations, with a marked acceleration since 2008. The United States dominated global production (1362 publications, 47.51%) and academic impact. The University of Pennsylvania was the most prolific institution, and Riegel B was the leading author. Keyword co-occurrence analysis identified four interconnected thematic domains: barriers to self-care and their prognostic impact, psychosocial factors and patient-reported outcomes, intervention strategies and healthcare delivery, and caregiver burden and dyadic interaction. Digital health, particularly telemedicine and mobile health, has emerged as a rapidly evolving frontier. **Conclusions:** HF self-care research continues to grow rapidly, with future efforts focused on caregiver burden and dyadic dynamics, equitable digital health, and personalized management for older adults with multimorbidity and cognitive impairment to ultimately improve clinical outcomes and quality of life for patients with HF.

## 1. Introduction

Heart failure (HF) is a complex clinical syndrome representing the end-stage manifestation of a broad spectrum of cardiovascular diseases, characterized by impaired cardiac function resulting from structural or functional cardiac abnormalities [1,2]. As a major global public health concern, HF affects approximately 1% to 6% of the global population, accounting for more than 64 million individuals worldwide. Its prevalence is projected to continue rising, primarily driven by improved post-diagnostic survival rates and population aging [3]. Patients with HF commonly endure a heavy symptom burden, with five to ten concurrent symptoms reported in most cases, which lead to functional disability and significant declines in health-related quality of life [4]. Despite recent advances in the treatment and management of HF, mortality and hospitalization rates remain high [5]. The substantial healthcare expenditures attributable to these elevated rates of mortality and hospitalization place a heavy economic burden on healthcare systems globally [6].

The 2021 European Society of Cardiology (ESC) Guidelines for the Diagnosis and Treatment of Acute and Chronic Heart Failure state that adequate patient self-care is fundamental to effective disease management and that it plays a critical role in reducing unplanned hospital readmissions, lowering mortality, and improving health outcomes in patients with HF [7]. The World Health Organization [8] defines self-care as “the ability of individuals, families, and communities to promote health, prevent disease, maintain health, and cope with illness and disability, with or without the support of a healthcare provider”. Riegel’s theory conceptualizes HF self-care as a multidimensional process encompassing self-care maintenance, symptom perception, and self-care management. Specifically, self-care maintenance involves health-promoting behaviors, including diet, physical activity, smoking and alcohol consumption management, mental health regulation, medication adherence, and engagement with healthcare services; symptom perception entails the recognition and interpretation of symptoms, the monitoring of changes in physical or psychological status, and awareness of early warning signs; and self-care management refers to decision-making and behavioral actions in response to symptomatic changes, such as medication adjustment, seeking professional support, implementing coping strategies, and modifying daily activities [9]. Although a growing body of evidence has substantiated the benefits of self-care in HF [10,11,12,13], the global research landscape remains poorly characterized, particularly regarding publication trends, collaborative networks, and evolving research hotspots.

Bibliometrics is a literature research methodology grounded in the principles of scientometrics. By integrating quantitative statistical methods with multiple analytical techniques, including co-word analysis, social network analysis, and clustering analysis, it systematically traces the developmental trajectory of specific research themes, identifies field-specific hot topics and emerging trends, and objectively evaluates the academic influence and contribution of authors, journals, institutions, and countries in a given research domain [14]. Previous bibliometric studies on HF have covered a wide range of research directions, including pathogenesis mechanisms (e.g., exosomes and gut microbiota), clinical therapeutics (e.g., pharmacotherapy and cardiac transplantation), common comorbidities (e.g., depression and sarcopenia), and innovative technological applications (e.g., artificial intelligence) [15,16,17,18,19,20,21]. Nevertheless, no bibliometric analysis to date has specifically focused on HF self-care. To address this research gap, the present study adopted bibliometric methods to systematically map the developmental trajectory of HF self-care research, aiming to provide a solid foundation for subsequent investigations and guide patient-oriented clinical decision-making.

To fill this gap, the present study addresses the following research questions:

(1) What are the overall publication and citation trends in HF self-care research over time?
(2) Which countries, institutions, authors, and journals constitute the most productive and influential contributors, and what are the collaborative patterns among them?
(3) What are the major research hotspots and thematic clusters, and what are the emerging trends and future directions?

## 2. Materials and Methods

### 2.1. Data Sources

Data on HF self-care research were retrieved from the Web of Science Core Collection (WoSCC), with the retrieval time frame spanning from the inception of the database to 29 June 2026. The WoSCC was selected for this bibliometric analysis due to its comprehensive coverage of high-quality, peer-reviewed journals across multiple disciplines [22]. Featuring powerful retrieval functions, this database allows for the efficient acquisition of extensive scientific literature and contributes to a holistic understanding of the knowledge structure in a specific research field.

The search strategy was formulated as follows: TS = (self care OR self-care OR self administration OR self medication OR self management OR self-management OR self-monitoring OR symptom perception OR self-regulation OR self management program OR self-management program OR self-care maintenance OR self-care management) AND TS = (heart failure OR HF OR cardiac failure OR heart decompensation OR myocardial failure OR right sided heart failure OR left sided heart failure OR congestive heart failure OR acute heart failure OR chronic heart failure).

**Figure 1:**
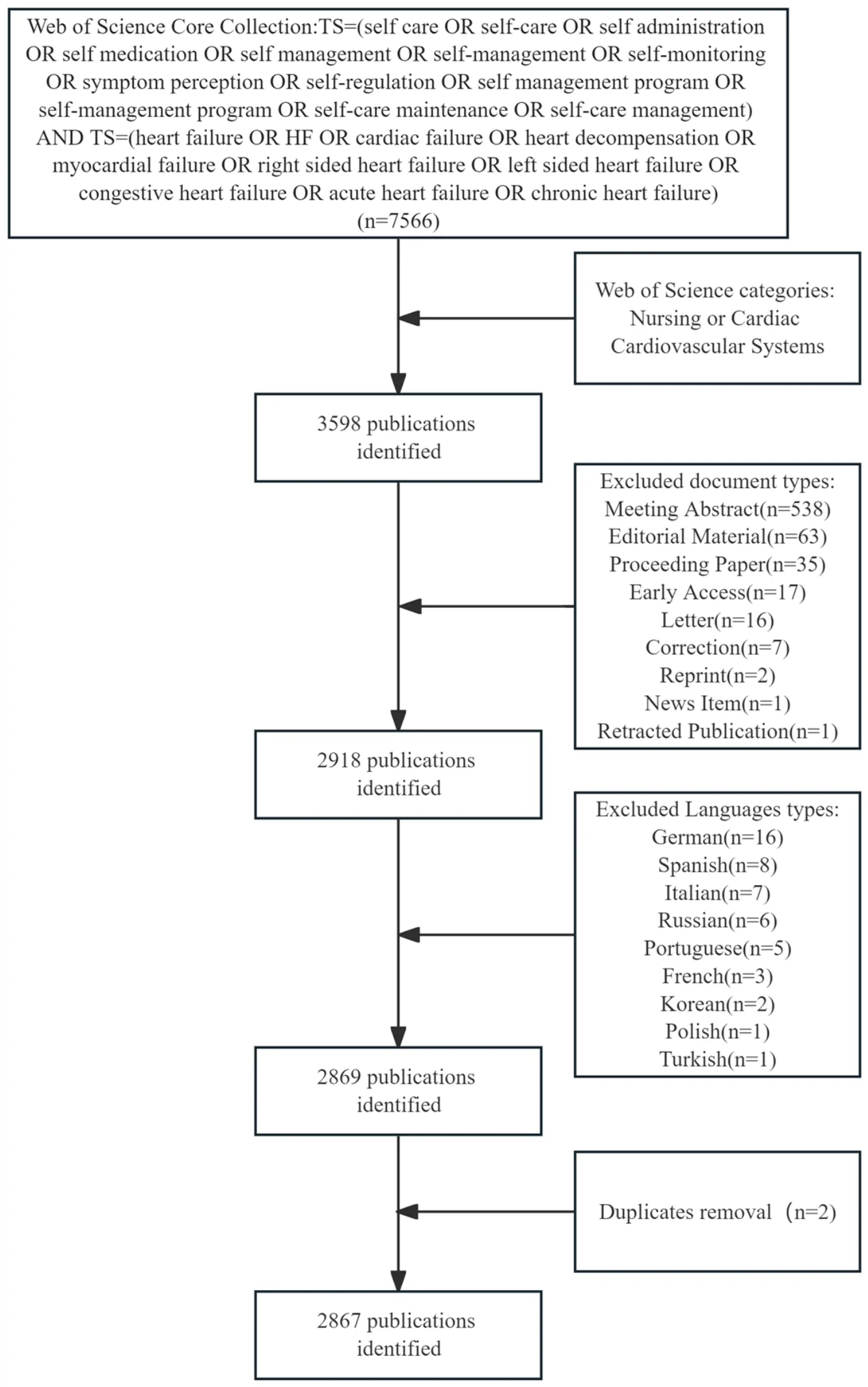
Flowchart for search process.

To further refine the research scope, the search was filtered according to Web of Science categories (“Nursing” or “Cardiac & Cardiovascular Systems”), publication types (“Article” or “Review”), and language (English). After duplicate removal, a total of 2867 unique records were obtained. To minimize potential bias resulting from daily database updates, all literature was exported on a single day (29 June 2026). The detailed search and screening procedure is illustrated in Figure 1.

### 2.2. Statistical Analysis

VOSviewer (version 1.6.20), a bibliometric analysis tool developed by Van Eck and Waltman from Leiden University (Leiden, The Netherlands) [23], extracts core metadata from publications and is extensively applied to construct collaboration networks, co-citation networks, and keyword co-occurrence networks [24]. Within VOSviewer, the total link strength quantifies the connection count of each node; every node stands for an analytical unit such as countries, institutions, authors, keywords, and co-cited references. Node size and node color separately denote quantitative metrics and group classification attributes, while the thickness of connecting lines mirrors the intensity of collaborative or co-citation associations between distinct entities [25]. We used full counting for all VOSviewer analyses.

CiteSpace (version 6.4.R1), a Java-based bibliometric visualization tool developed by Chaomei Chen from the College of Computing and Informatics, Drexel University (Philadelphia, PA, USA), enables visual mapping of citation networks and identification of thematic research hotspots [24]. The time slicing window was set to span January 1980 to December 2026, with each slice covering one calendar year. Link strength was calculated via cosine similarity within individual time slices. Nodes were filtered using the g-index with a scaling factor k = 7. Pathfinder pruning and sliced network pruning were implemented to simplify network topology and retain core inter-node connections, whereas minimum spanning tree pruning and merged network pruning were excluded from the analytical pipeline.

Microsoft Excel 2019 (Microsoft Corporation, Redmond, WA, USA) was utilized for data organization, sorting, and statistical analysis of publication outputs. Detailed journal classification information was retrieved from the 2025 Journal Citation Reports (JCR). The H-index, originally proposed by Hirsch in 2005, is defined as the maximum h value, indicating that a research entity has published h papers, each of which has received no fewer than h citations [26].

A manually curated thesaurus file was adopted to standardize heterogeneous expression variants across the retrieved literature, including conceptual synonyms, hyphenated and spaced spellings, singular and plural forms, professional abbreviations, institutional aliases, and geographical name inconsistencies (e.g., “heart-failure” vs. “heart failure”, “turkiye” vs. “turkey”). All term mappings in the thesaurus file were manually verified to ensure analytical accuracy, with detailed merging rules provided in the Supplementary Materials.

## 3. Results

### 3.1. Publication and Citation Trends

From database inception to 29 June 2026, a total of 2867 publications on HF self-care were indexed in the WoSCC, comprising 2500 articles and 367 reviews. These publications received 97,346 citations (79,252 excluding self-citations), with an H-index of 126.

**Figure 2:**
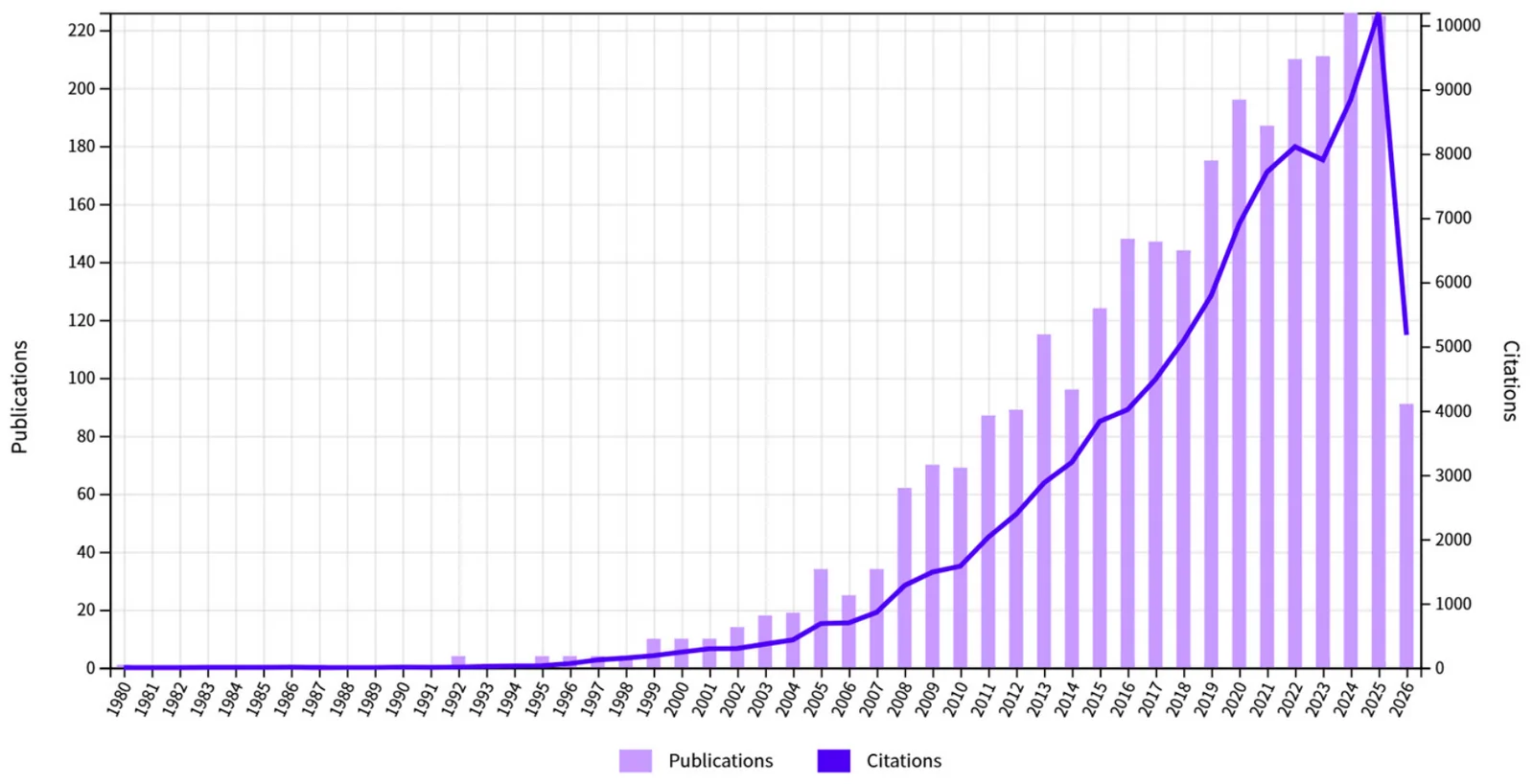
Publication trends in HF self-care research.

In terms of annual publication trends, the first scholarly article related to HF self-care was published in 1980. Between 1980 and 1998, the annual number of publications in this field remained consistently below 10. Annual publication output first surpassed 10 in 1999, representing an initial rise in research activity in this domain. Both publication quantity and citation frequency increased steadily and moderately from 1999 to 2007. Since 2008, the research field has entered a phase of rapid and sustained growth. The annual publication volume reached a peak of 226 in 2024 and maintained a relatively high level in 2025. Notably, the 2026 dataset only covered literature published up to late June, which cannot reflect the full-year publication trend for 2026. The temporal citation trend was largely consistent with the growth trajectory of publications. Annual citations experienced a slight decrease from 2022 to 2023 but rose markedly from 2023 to 2025, suggesting a continuous enhancement of the academic influence of HF self-care research in recent years (Figure 2). Overall, both annual publication volume and total citation counts showed a persistent upward trend from 1980 to 2026, indicating growing scholarly attention and expanding academic impact of this research area.

### 3.2. Contributions of Countries and Institutions

A total of 95 countries and regions contributed publications to the field of HF self-care from the inception of the database to 29 June 2026. The top 10 countries ranked by total publication volume are listed in Table 1. The United States (USA) dominated the global research output and academic influence in this field, with 1362 publications (47.51%), an average citation of 39.87 per article, and an H-index of 104. The USA substantially outperformed other nations in both publication quantity and citation impact, maintaining a leading position in HF self-care research. Sweden ranked second with 239 publications (8.34%), followed by Australia with 232 publications (8.09%). China occupied the fourth position with 229 publications (7.99%), whereas its average citations (13.86) and H-index (29) remained relatively low compared with other top-ranked countries. The other countries within the top 10 list produced 108 to 227 publications with H-indices ranging from 24 to 59, revealing prominent disparities in academic influence among different nations worldwide.

**Figure 3:**
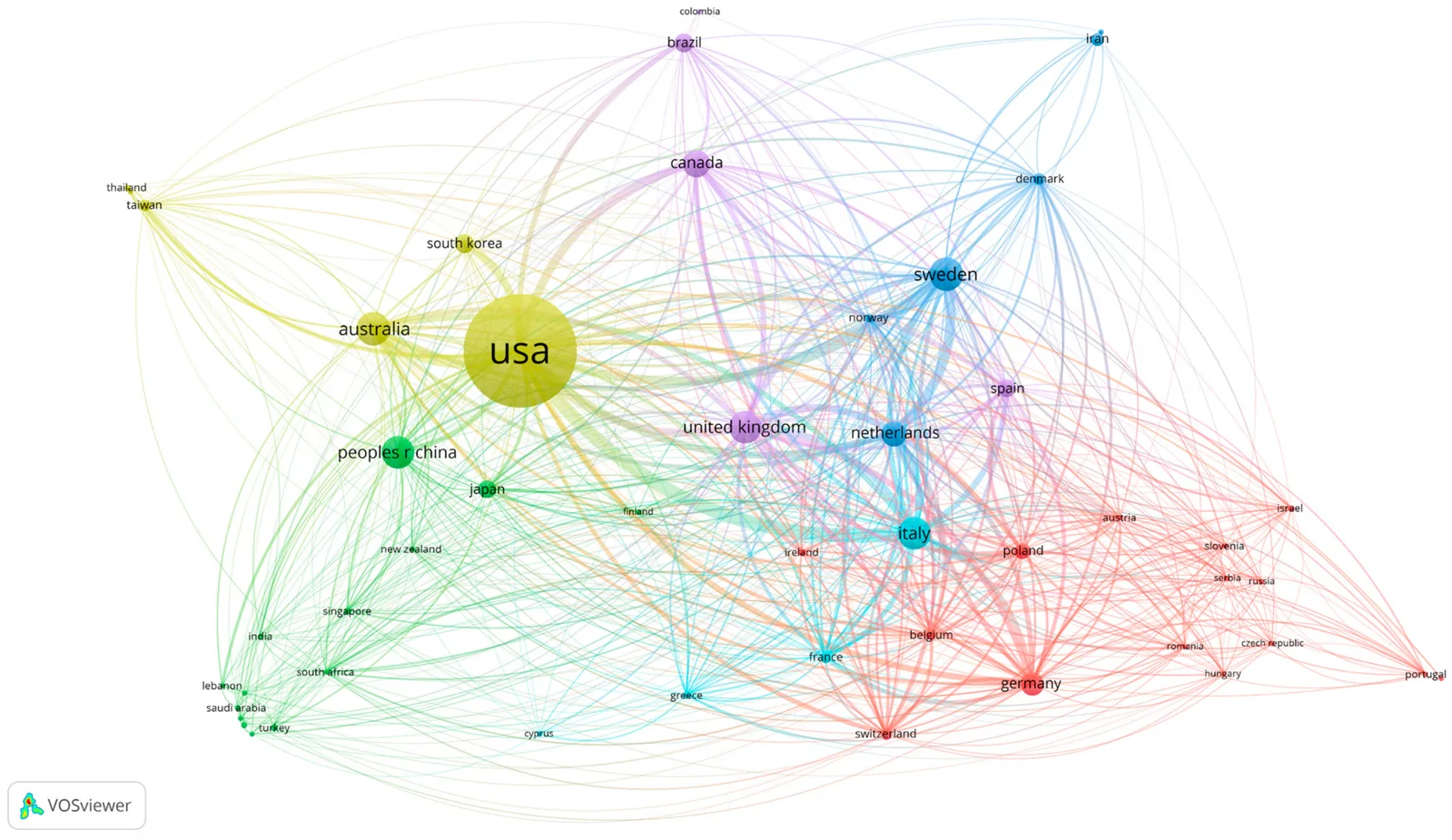
Cooperative network of countries with more than 8 publications. Note: Each node represents a country, with node size reflecting the number of publications, node color denoting different clusters, and line thickness indicating the strength of collaborative relationships between countries.

Furthermore, international collaborative patterns were visualized via VOSviewer, with a publication threshold of more than 8 publications for country inclusion. The co-occurrence network analysis indicated that the USA, the United Kingdom, Italy, Sweden, the Netherlands, and Germany constituted the core contributing countries in this research field. The USA achieved the highest total link strength of 803, demonstrating its central status and dominant collaborative role in the global HF self-care research network. Australia and Italy maintained the closest cooperative ties with the USA (Figure 3).

**Figure 4:**
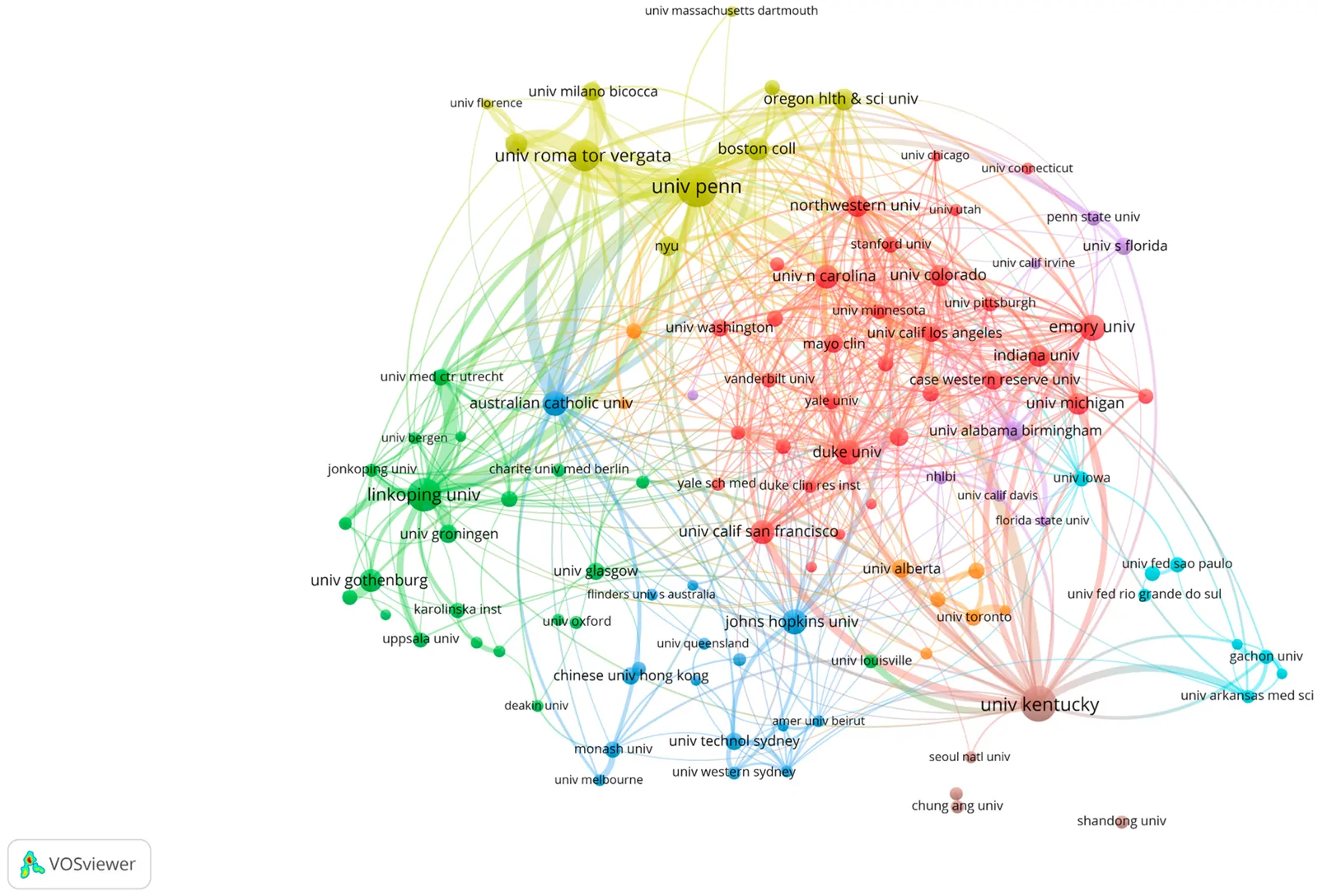
Cooperative network of institutions with more than 13 publications. Note: Each node represents an institution, with node size reflecting the number of publications, node color denoting different clusters, and line thickness indicating the strength of collaborative relationships between institutions.

A total of 3906 institutions contributed publications to the HF self-care literature from database inception to 29 June 2026. The top 10 institutions ranked by publication volume are presented in Table 2. The University of Pennsylvania ranked first with 177 publications and possessed the highest H-index (64) among the top 10 institutions, demonstrating its leading academic influence within this research field. Geographically, seven of the top 10 institutions were located in the USA, while the remaining three were affiliated with Sweden, Italy, and Australia, respectively. Notably, Linköping University independently published 115 articles, accounting for 48.12% of Sweden’s total publications in this domain, which underscored its dominant position in the country’s HF self-care research output. Figure 4 visualizes the collaborative network of institutions with more than 13 publications. The University of Pennsylvania yielded the highest total link strength (357) and maintained the closest cooperative ties with the University of Rome Tor Vergata. Additionally, the University of Kentucky, Linköping University, Australian Catholic University, and Duke University exhibited prominent collaborative activity. Collectively, these core institutions formed a tight collaborative network that facilitated the advancement of HF self-care research.

### 3.3. Contributions of Authors and Journals

**Figure 5:**
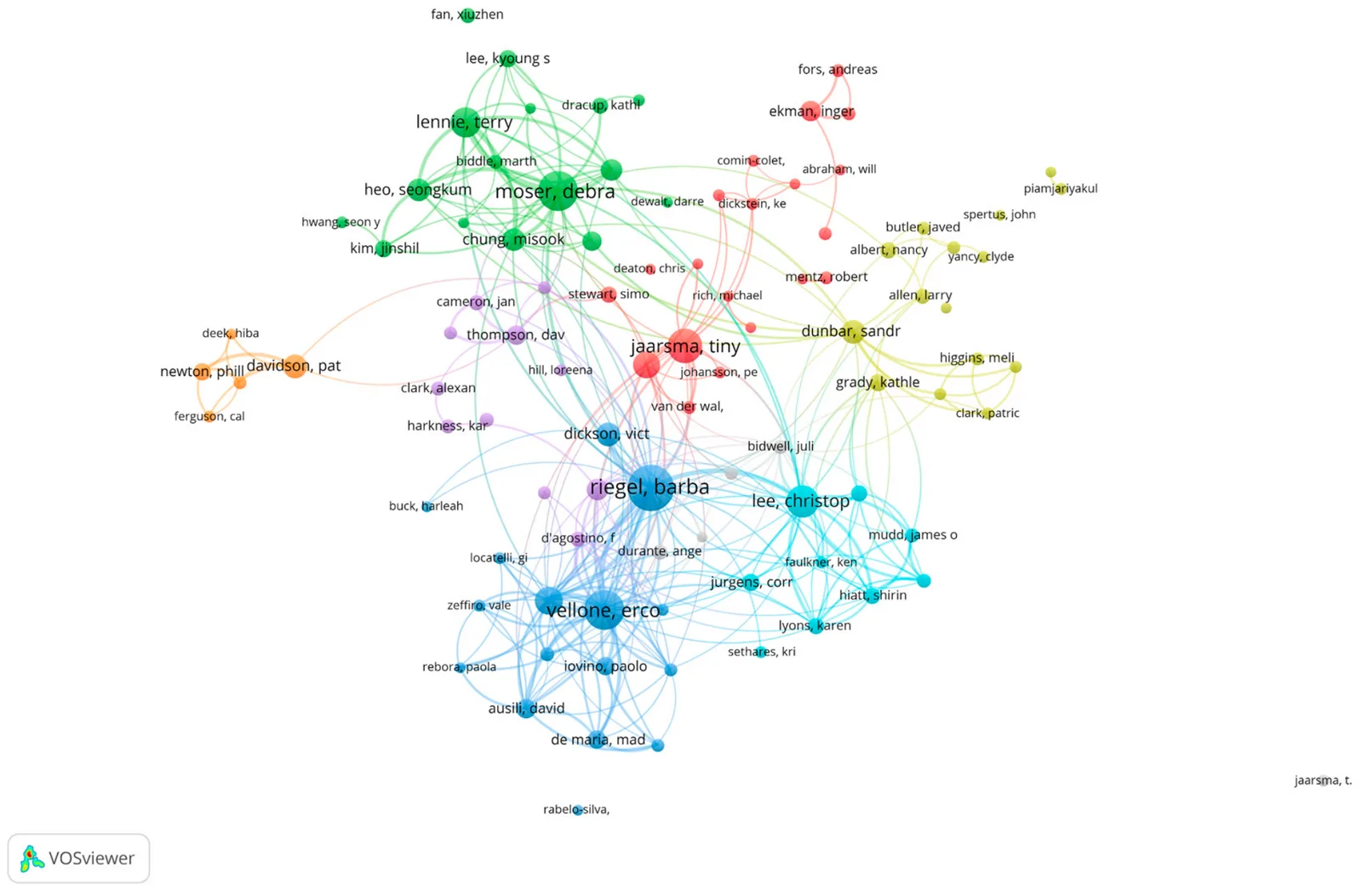
Co-occurrence diagram of authors with more than 9 publications. Note: Each node represents an author, with node size reflecting the number of publications, node color denoting different clusters, and line thickness indicating the strength of collaborative relationships between authors.

A total of 12,119 authors contributed to HF self-care publications indexed in the WoSCC from database inception to 29 June 2026. The top 10 authors ranked by publication volume are presented in Table 3. Riegel B from the University of Pennsylvania was the most influential author, with 135 publications, 10,730 citations, and an H-index of 63, underscoring her central role in shaping this research field. Figure 5 illustrates the collaborative network of authors with more than 9 publications. The co-authorship network revealed that Riegel B, Vellone E, Moser DK, Jaarsma T, Lee CS, and Lennie TA constituted the core scholarly community in this field, with dense collaborative ties among them.

A total of 278 journals published HF self-care research. Table 4 lists the top 10 journals ranked by publication volume, along with their 2025 JCR quartile information. The Journal of Cardiovascular Nursing (*n* = 308) ranked first, followed by the European Journal of Cardiovascular Nursing (*n* = 227). The remaining journals each contributed between 55 and 170 publications. Notably, six of these ten journals (60%) are classified as Q1 journals based on the 2025 JCR, reflecting their strong academic influence, while the other four are Q2 journals. Journal co-citation analysis can reveal academic connections and the strength of knowledge linkages among different journals. A total of 13,639 journals cited the retrieved HF self-care publications. Table 5 presents the top 10 co-cited journals in this field, eight of which are categorized as Q1 in the 2025 JCR. The Journal of Cardiovascular Nursing topped the list with 5040 co-citations, while Circulation achieved the highest total link strength (80,499). The remaining journals yielded co-citation counts ranging from 1754 to 4022 and total link strengths between 31,960 and 68,569, acting as pivotal knowledge nodes in the HF self-care research network.

### 3.4. Keywords Analysis of Clusters and Bursts

Keyword co-occurrence analysis facilitates the rapid identification of research hotspots in a specific research domain. A total of 6467 keywords were extracted from the titles and abstracts of all included publications via VOSviewer. Table 6 lists the top 20 keywords ranked by occurrence frequency. The most frequently occurring term was “heart failure” (1862), followed by “self-care” (1104), “quality of life” (754), “management” (623), and “outcomes” (432).

**Figure 6:**
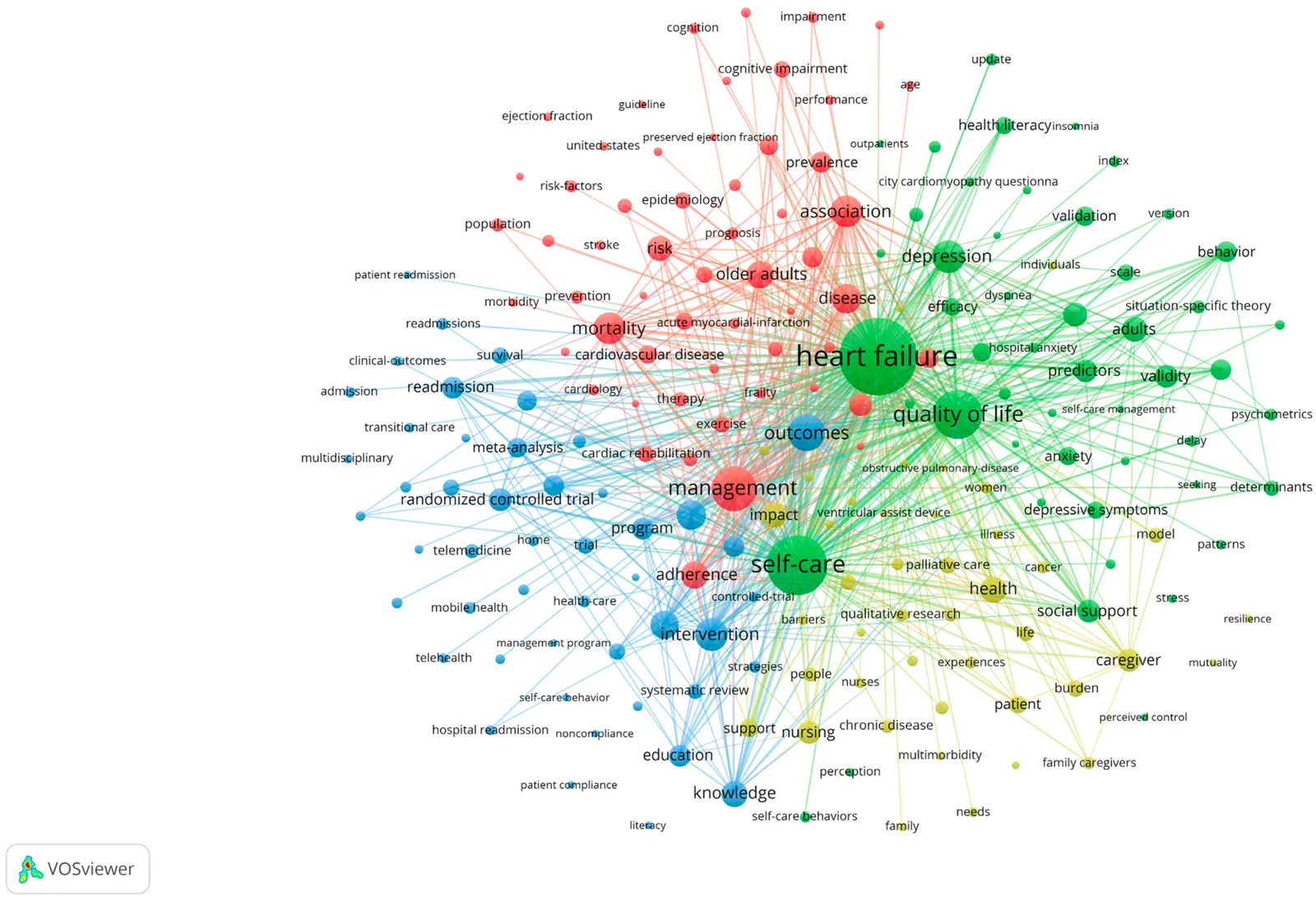
Co-occurrence network of keywords in HF self-care research. Note: Each node represents a keyword, with node size reflecting the occurrence frequency, node color denoting different clusters, and line thickness indicating the strength of co-occurrence relationships between keywords.

**Figure 7:**
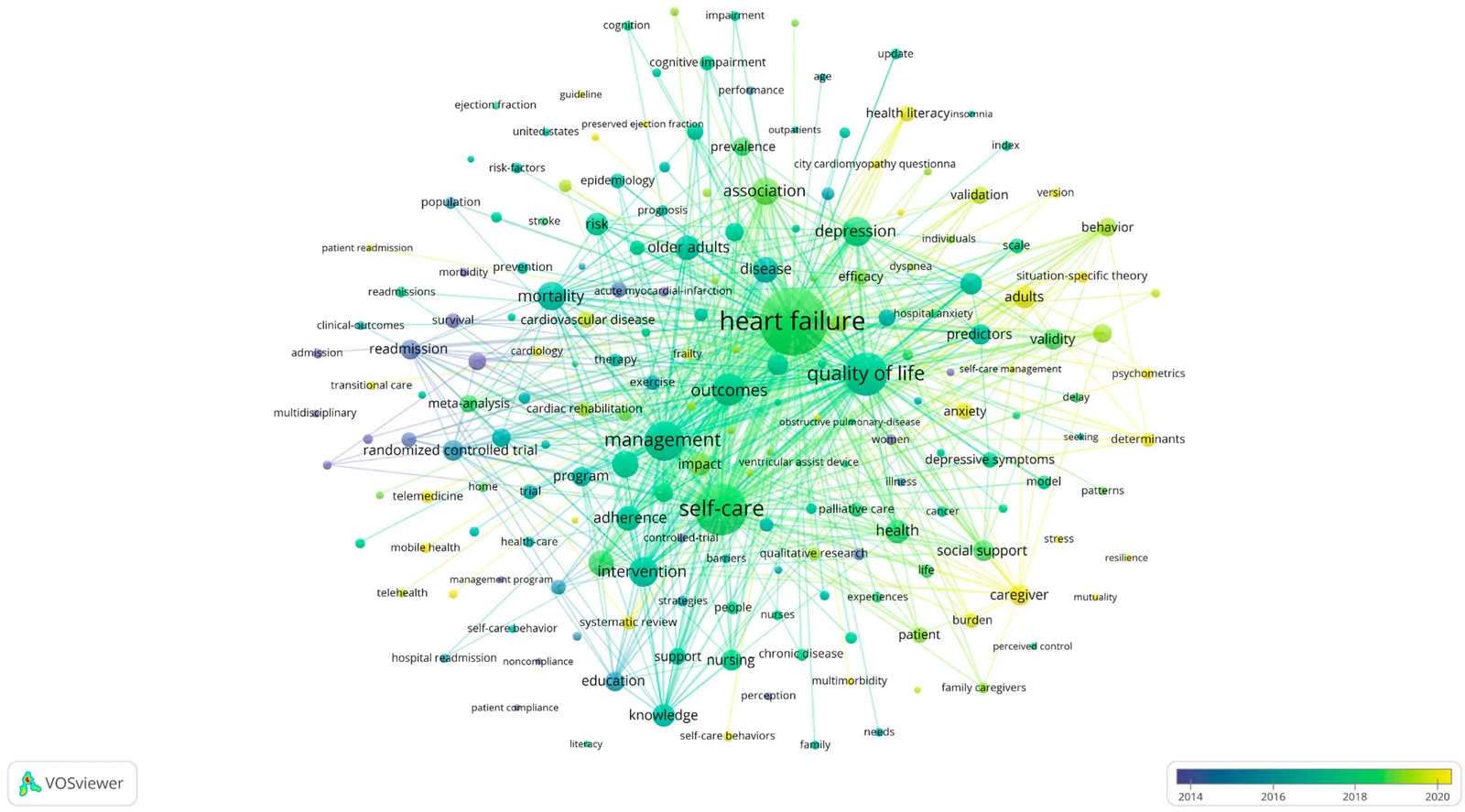
The average publication years of the keywords. Note: Node color represents the average publication year, with blue indicating earlier years and yellow indicating more recent years.

Keyword co-occurrence analysis was performed via VOSviewer, focusing on keywords with a minimum occurrence of 22 (Figure 6). The results indicated that “heart failure” and “self-care” served as the core research themes in this domain. Clusters in different colors represented distinct research directions within HF self-care research. The red cluster contained closely interconnected keywords, including “management”, “association”, “mortality”, “disease”, “older adults”, “adherence”, “risk”, “cardiovascular disease”, “prognosis”, “risk-factors”, and “cognitive impairment”. The green cluster revealed tight correlations between “heart failure” and multiple key terms, namely “self-care”, “quality of life”, “depression”, “symptoms”, “social support”, “validity”, “anxiety”, and “depressive symptoms”. The blue cluster covered interrelated keywords centered on “outcomes”, “intervention”, “care”, “self-management”, “knowledge”, “randomized controlled trial”, “program”, “patient education”, “readmission”, “medication adherence”, “systematic review”, and “telemedicine”. The yellow cluster mainly focused on “health”, “impact”, “nursing”, “caregiver”, “support”, “patient”, “burden”, “quality”, “life”, “family caregivers”, “perceptions”, “experiences”, and “mutuality”. The average publication years of these keywords are visualized in Figure 7.

**Figure 8:**
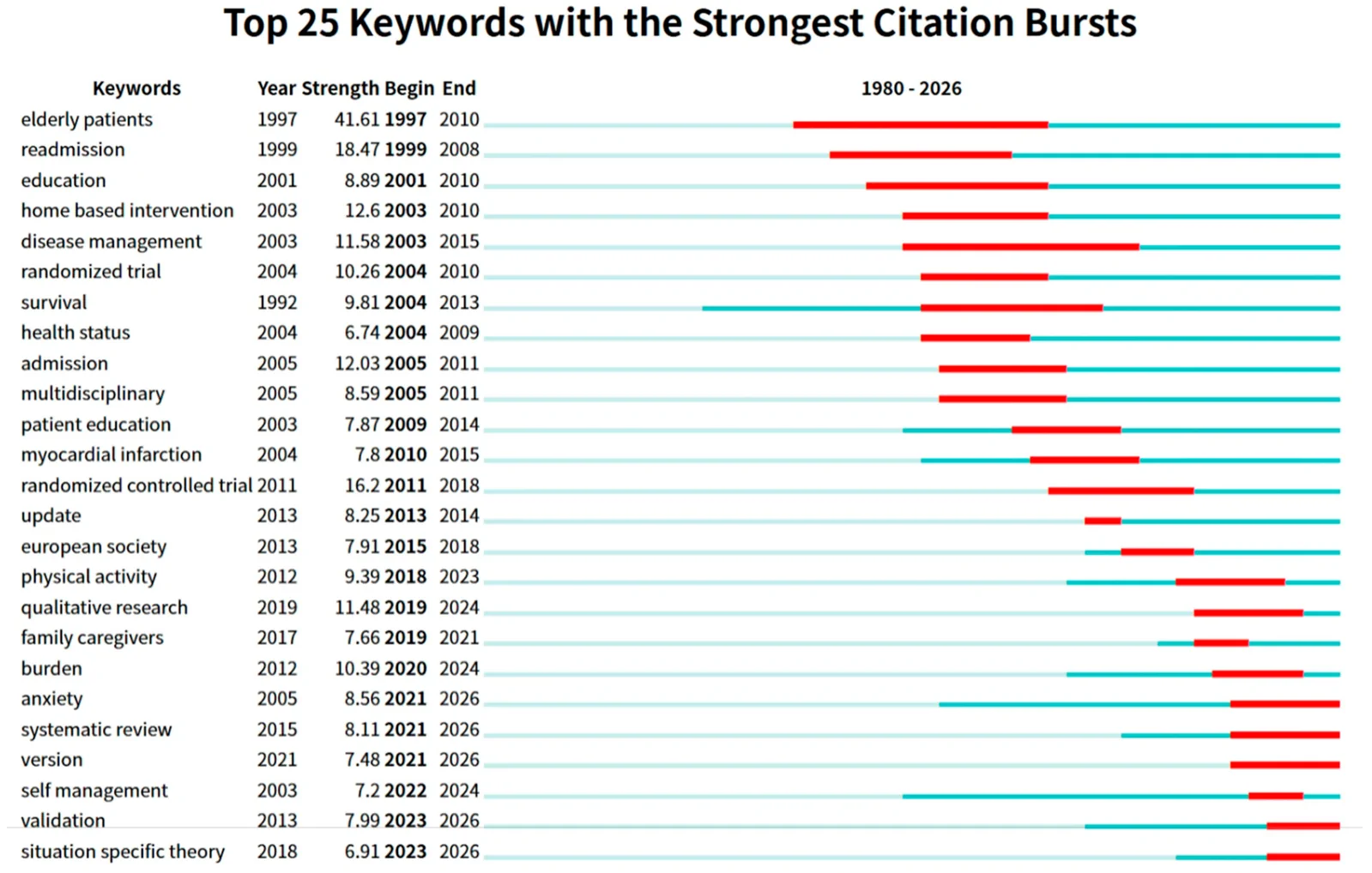
Top 25 Keywords with the Strongest Citation Bursts.

Keyword burst analysis was further conducted to identify dynamic research hotspots that received extensive scholarly attention and citations across different periods (Figure 8). The red line segments represent the duration of sustained high-frequency occurrence of individual keywords, while the strength value reflects the burst intensity of each term. In recent years, the keywords with the strongest burst intensity in the field of HF self-care research included “anxiety”, “systematic review”, “version”, “validation”, and “situation specific theory”.

### 3.5. Analysis of Co-Cited References

**Figure 9:**
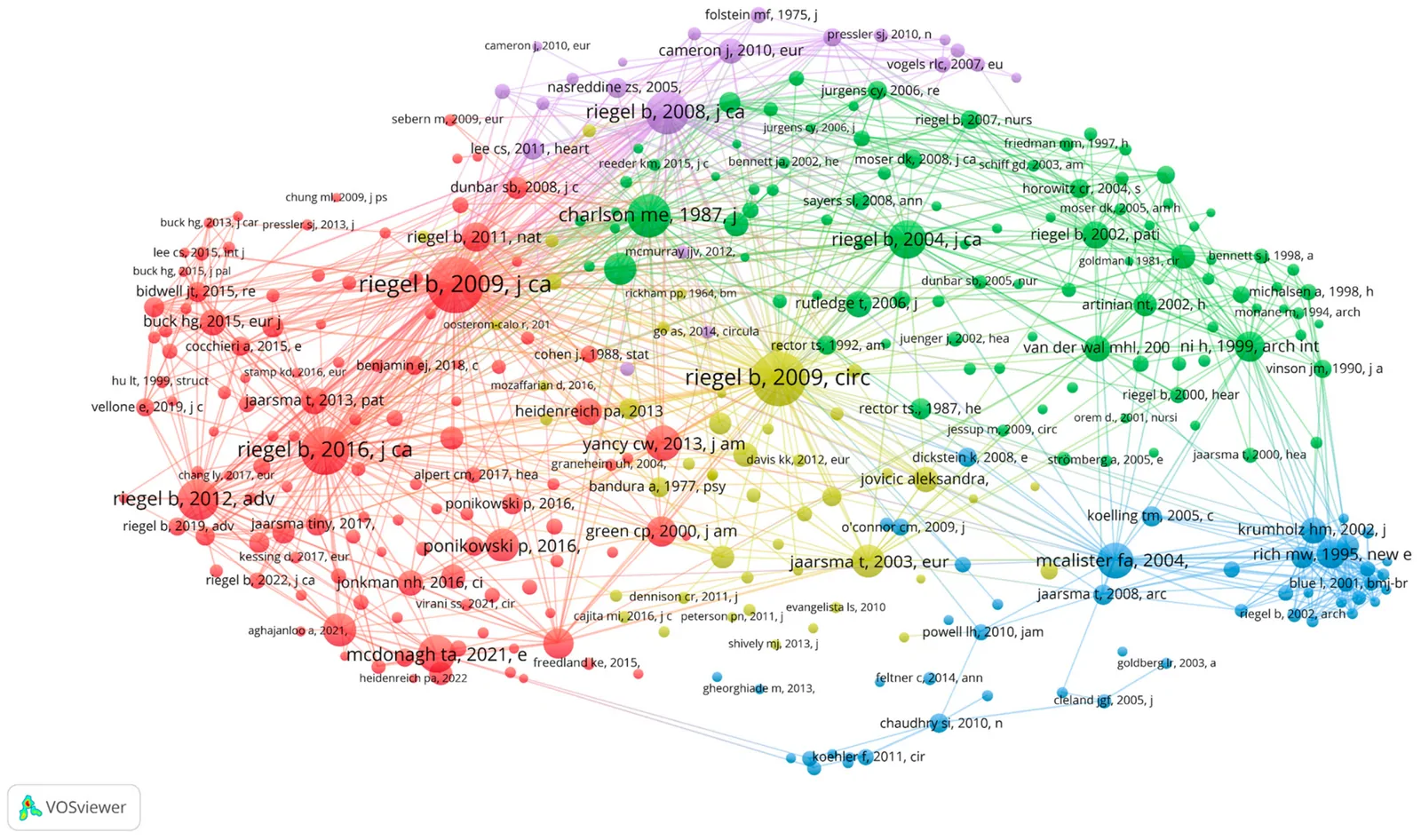
Co-occurrence network of co-cited references in HF self-care research. Note: Each node represents a co-cited reference, with node size reflecting the co-citation frequency, node color denoting different clusters, and line thickness indicating the strength of co-citation relationships between references.

VOSviewer was used to screen references with a co-citation frequency of ≥20 for network co-occurrence analysis, and five collaborative clusters were identified in the global research network (Figure 9). The core cited references mainly consisted of landmark studies published by Riegel B in 2008 and 2009, as well as a classic study published by Mcalister FA in 2004. Among the top 10 co-cited references, six were authored by Riegel B, which further confirmed her pivotal academic status in the field of HF self-care research. The research hotspots of co-cited literature primarily focused on HF self-care theories, methodological validation, and clinical practice guidelines. The most highly co-cited publication was a 2009 study by Riegel B et al. published in the Journal of Cardiovascular Nursing (276), which conducted an updated psychometric validation of the Self-Care of Heart Failure Index. The second-most co-cited work was a 2009 scientific statement from the American Heart Association published in Circulation by Riegel B et al. (265), which provided standardized recommendations for optimizing self-care practices among patients with HF.

**Figure 10:**
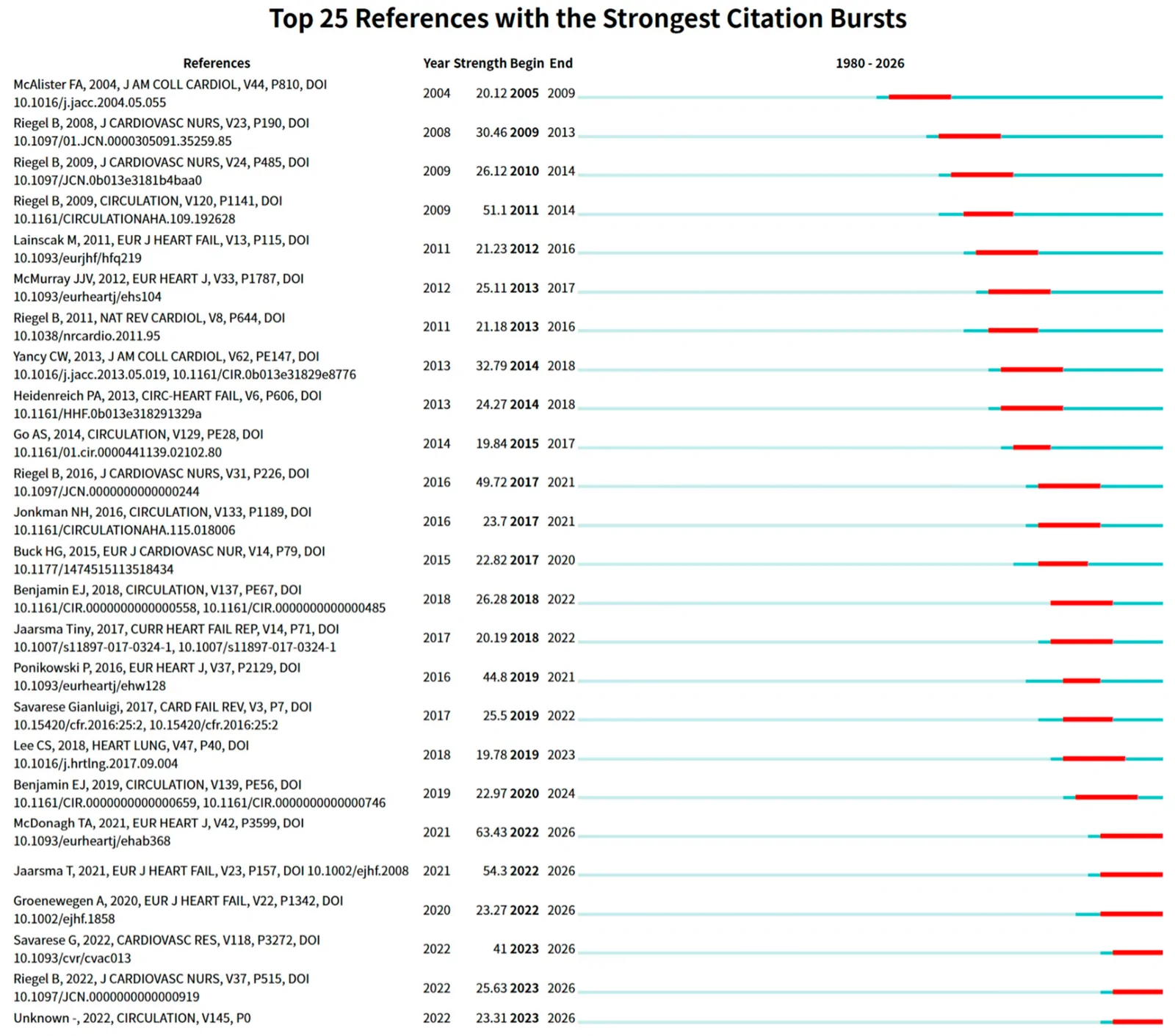
Top 25 References with the Strongest Citation Bursts.

Co-citation burst detection identifies publications or authors that have received widespread attention during specific periods (Figure 10). The red line segments indicate the duration of high-frequency occurrence of the co-cited references, while the “strength” value represents the burst intensity of the co-cited references. Burst detection revealed that two articles exhibited high burst strength: the 2021 article by McDonagh TA et al. [7], titled “2021 ESC Guidelines for the Diagnosis and Treatment of Acute and Chronic Heart Failure”, and the 2021 article by Jaarsma T et al. [27], titled “Self-Care of Heart Failure Patients: Practical Management Recommendations from the Heart Failure Association of the European Society of Cardiology”. This indicated that the HF treatment guidelines developed by the ESC and the patient self-management recommendations garnered significant attention from researchers. Concurrently, studies by Groenewegen A et al. [28] and Savarese G et al. [3] on the epidemiology and burden of HF, and the latest theoretical update by Riegel B et al. [29], also demonstrated significant burst activity. This update highlights the interplay of problem, person, and environmental factors in HF self-care, calling for more attention to underexplored areas.

### 3.6. Analysis of Funding Agencies

This study analyzed the top 10 funding agencies for global HF self-care research based on publication volume (Table 7). The results indicated a striking regional imbalance in research funding, with USA institutions dominating the field. The United States Department of Health and Human Services and the National Institutes of Health served as the primary funding sources, while the National Heart, Lung, and Blood Institute and the National Institute of Nursing Research provided sustained support for cardiovascular and nursing-focused HF self-care studies. By comparison, funding agencies from South Korea and Japan contributed far fewer publications, demonstrating that global HF self-care research funding is highly concentrated in the USA.

## 4. Discussion

### 4.1. General Information

This study analyzed 2867 HF self-care publications authored by 12,119 scholars and published in 278 WoSCC-indexed journals from 1980 to 2026. Bibliometric analyses using CiteSpace and VOSviewer confirmed a sustained annual increase in publication and citation outputs in this field. HF self-care research achieved rapid and robust development since 2008, with annual publications peaking at 226 in 2024 and maintaining a high volume in 2025. This critical developmental turning point was mainly driven by the landmark situation-specific theory of heart failure self-care established by Riegel B et al. [30] in 2008. As a foundational theoretical framework, this theory delineates two core components of HF self-care (self-care maintenance and self-care management) and clarifies the regulatory impact of self-care confidence on clinical outcomes. With empirically validated theoretical propositions, this framework unifies the conceptual definition of HF self-care and standardizes research paradigms, greatly promoting advances in global HF self-care research. In addition, the continuous iteration and widespread dissemination of international authoritative guidelines and professional consensus, including practical self-care recommendations from the Heart Failure Association of the ESC, have further recognized and standardized the value of self-management in holistic HF treatment [7,27,31], collectively fostering the long-term and sustainable development of this research field.

The USA consistently ranks first in total publications, citation counts, H-index, and total link strength in HF self-care research. This finding demonstrates that the USA not only yields abundant high-quality research outputs in this field but also serves as a core hub for advancing international academic collaboration. Its leading research status can be primarily attributed to two key factors. On the one hand, the USA sustains long-term, stable, and high-intensity research funding support. Authoritative institutions including the National Institutes of Health continuously invest in basic research and clinical translational studies related to HF self-care, providing solid financial guarantees for high-yield and high-quality academic achievements. On the other hand, the pioneering and iteratively updated core theories in this domain, represented by Riegel’s HF self-care theory, were initially proposed and progressively optimized by American academic institutions [9,29,30]. These theoretical advances have established a dominant theoretical foundation and unified research framework for global studies, guiding the directional development of subsequent research worldwide. Although China ranks fourth globally in publication output, its relatively low average citations per article (13.86) and limited total link strength (138) reflect weaker academic influence and less-intensive international collaboration relative to leading high-output countries. This bibliometric gap does not equate to poor quality research but may be explained by two primary contextual factors. For one thing, language barriers put Chinese researchers at a disadvantage in the English-dominated global academic landscape, restricting the international visibility and citation potential of their work [32]. Furthermore, HF self-care research in China began substantially later than in Western countries; 92.14% of its English-language publications have emerged over the past decade, allowing limited time for citations to accumulate. Nevertheless, Chinese studies provide valuable real-world evidence from East Asian patient populations, complementing the largely Western-centered evidence base. Future research should foster international collaborative partnerships, optimize academic writing for global audiences, and promote the dissemination of region-specific findings to enhance the international profile of Chinese HF self-care research.

Institutional collaborative analysis further demonstrates that the University of Pennsylvania ranks first in both publication volume and total link strength, occupying a dominant hub within the global HF self-care collaborative network. Notably, Linköping University and the University of Rome Tor Vergata account for 48.12% of Sweden’s and 46.88% of Italy’s HF self-care publications, respectively. This observation underscores that high-impact regional institutions substantially shape national research competitiveness and drive disciplinary advancement. The bibliometric author analysis identifies Riegel B as the most prolific and frequently cited scholar in the field. As the originator of the situation-specific theory of HF self-care, she has fundamentally shaped the theoretical, methodological, and clinical landscape of this domain through theoretical construction [9,30,33,34,35], measurement tool development [36,37,38], and clinical guideline formulation [27,39,40]. Journal distribution analysis indicates that the Journal of Cardiovascular Nursing ranks first with 308 publications, closely followed by the European Journal of Cardiovascular Nursing with 227 publications. As core journals focusing on cardiovascular nursing, they act as primary outlets for disseminating pioneering HF self-care findings and play a pivotal role in facilitating academic communication and clinical knowledge exchange within this research field.

### 4.2. Research Hotspots in HF Self-Care

Through the keyword co-occurrence network and the strongest keyword burst analysis, the research hotspots and trends in this field were revealed. In this study, 4 clusters emerged from the keyword co-occurrence network. These can be summarized as four major thematic domains: barriers to self-care and their prognostic impact, psychosocial factors and patient-reported outcomes, intervention strategies and healthcare delivery, and caregiver burden and dyadic interaction.

Current guidelines have recognized the critical role of self-care in reducing unplanned hospital readmissions, lowering mortality, and improving health outcomes in patients with HF [7,41]. However, self-care engagement remains suboptimal among patients with HF [42], with only 7.6% to 18% reporting adequate self-care behaviors [43]. According to the updated situation-specific theory of heart failure self-care, self-care is a naturalistic decision-making process shaped by the dynamic interaction of three categories of factors: problem-related (e.g., cognitive impairment, depression, and symptom burden), person-related (e.g., age, health literacy, perceived control, and self-efficacy), and environmental (e.g., social support, access to care, and built environment) [29]. The 2009 American Heart Association scientific statement on promoting self-care in persons with HF explicitly recognizes that comorbidities such as “hypertension,” “coronary heart disease,” and “cardiovascular disease” substantially complicate disease management and pose significant barriers to effective self-care [39]. These conditions necessitate complex pharmacological regimens and two or more conflicting dietary requirements, directly undermining “adherence” and complicating self-care decision-making [39]. Moreover, the statement highlights that “older adults” with HF frequently present with “cognitive impairment,” which erodes the memory, attention, and executive function required for daily self-care maintenance and symptom interpretation [39]. Consequently, multimorbidity compounded by advanced age and cognitive impairment not only impedes self-care engagement but also elevates “mortality” risk and worsens “prognosis,” underscoring the necessity of tailored management.

The emergence of “quality of life”, “depression”, “anxiety”, “symptoms”, and “social support” indicates that the research scope has expanded beyond traditional clinical endpoints, with research focus shifting toward patients’ subjective illness experience and psychosocial adaptation. HF symptoms and treatment-related outcomes greatly affect the lives of HF patients; therefore, improving quality of life is generally recognized as one of the major goals of HF treatment [44]. Notably, HF patients’ quality of life is shaped by a constellation of interrelated factors, among which symptom burden constitutes one of the most prominent determinants of patients’ quality of life [45]. HF patients commonly suffer from overlapping physical manifestations, including dyspnea, fatigue, insomnia, and peripheral edema, which collectively restrict daily functional performance and undermine subjective comfort and general well-being [46,47], thereby diminishing their overall quality of life. Accordingly, interventions targeting these symptoms can effectively improve the quality of life of patients with HF [48]. In accordance with the practical management recommendations issued by the Heart Failure Association of the ESC, self-care is closely linked to person-centered outcomes among heart failure patients, particularly enhanced quality of life [27]. A growing cohort of scholars has therefore directed sustained research attention to the facilitating effect of standardized HF self-care behaviors on the elevation in patients’ quality of life, verifying stable positive correlations between the two variables via cross-sectional, longitudinal and interventional research designs [49,50]. Psychosocial factors including anxiety, depression and social support also mediate the association between self-care and quality of life, consistent with the keyword distribution observed in this study. Patients with HF frequently experience prominent psychological distress, with anxiety and depression reported to affect 53.4% and 52.4% of this population, respectively [51]. Depression is a core symptom during the vulnerable phase of HF. Both anxiety and depression not only reduce self-care adherence but also exacerbate their symptom burden [52]. Additionally, patients’ perceived social support may exert a positive influence on their self-care behaviors, including symptom management and adherence to HF-related treatment regimens [53,54]. Past research confirms that anxiety and depression are independent predictors of poorer health-related quality of life among HF patients [55,56]. Meanwhile, social support plays a protective role: it relieves psychological distress caused by negative emotions, and indirectly improves quality of life through mediating depressive symptoms [57,58]. Clinical expert recommendations advise routine screening for anxiety, depressive symptoms and social support needs to improve HF patients’ quality of life [27].

The blue cluster reflects a sustained scholarly emphasis on intervention strategies and healthcare delivery models designed to enhance self-management behaviors and improve clinical outcomes among patients with HF. As reflected in this cluster, current self-care interventions can be broadly classified into three categories: transitional care, home care, and remote interventions. Transitional care integrates in-hospital guidance with post-discharge follow-up via home visits, telephone calls, or digital platforms. Home care primarily involves regular home visits supplemented by telephone or digital monitoring. Remote interventions rely solely on telephone communication and digital medical platforms to deliver self-care guidance [59]. “Randomized controlled trials” have served as the predominant methodological framework for evaluating the efficacy of structured self-care programs, yielding robust evidence that self-management interventions improve patient survival and self-care adherence and reduce hospital readmissions and length of stay [44,60,61,62]. “Medication adherence” constitutes one of the core behaviors of HF self-care maintenance; however, polypharmacy regimens, complex dosing schedules, and suboptimal health literacy continue to compromise adherence rates in clinical practice [39,63,64]. Notably, the average publication year analysis of keywords identified “telemedicine” as a rapidly emerging research frontier, reflecting the accelerating integration of digital health technologies—including remote monitoring, mobile health applications, and teleconsultation platforms—into HF self-care delivery [65,66]. Furthermore, the prominence of “systematic review” among recent burst keywords underscores a maturing evidence base, as the field increasingly relies on synthesized evidence to inform guideline development and optimize intervention design.

The co-occurrence of the keywords “health”, “impact”, “nursing”, “caregiver”, “support”, “patient”, “burden”, “quality”, “life”, “family caregivers”, “perceptions”, “experiences”, and “mutuality” within this thematic cluster indicates that scholarly attention has gradually shifted toward caregiver burden and the dyadic interplay between patients and their caregivers. Indeed, family caregivers typically undertake substantial responsibilities in assisting patients with symptom management, self-care promotion, and health-related decision-making [67]. This support is associated with patient adherence to self-care recommendations and fewer hospitalizations [68]. Nevertheless, this supportive role is frequently accompanied by severe caregiver burden, depressive symptoms, and impaired quality of life among caregivers [69,70]. Caregiver burden refers to the multifaceted burden caregivers experience over time while caring for family members or loved ones [71]. Previous research has reported that 86.7% of HF caregivers experienced high levels of caregiver burden [72]. This burden may reduce the quality of care provided, leading to greater risks of readmissions and mortality for care recipients and higher burdens on caregivers [67,73]. Studies have emphasized that HF self-care is a dyadic phenomenon, wherein patient self-care influences caregiver contributions to self-care, and vice versa [35]. The HF experience is shared—to varying degrees—by both the patient and their caregiver. Thus, an important line of research involves characterizing interactions within the patient–caregiver dyad (i.e., the unit of interest is the relationship, rather than the individual patient or caregiver) [67]. Mutuality, defined as the degree of love and affection, shared activities, shared values, and empathy in the patient–caregiver relationship, has been described as a measure of relationship quality [74]. Moreover, evidence suggests that relationship quality is associated with lower risk of mortality, better health status, and reduced caregiver burden [75]. Also, better relationship quality appears to be associated with better patient self-care and caregiver contributions to patient self-care, although it is the individual’s own perception of relationship quality that matters [76]. Therefore, integrating caregiver burden reduction and dyadic relationship enhancement into HF self-care frameworks remains a critical imperative for future research and practice.

Building on the identified research hotspots and frontier dynamics, future developmental trends in HF self-care research are increasingly centered on digital health, as reflected by emerging keywords such as “telemedicine”, “mobile health”, “telehealth”, and “technology”. Driven by the global proliferation of mobile devices, digital technologies are fundamentally reshaping the landscape of self-care management, enabling remote monitoring, real-time symptom tracking, and personalized health coaching, directly addressing conventional constraints such as suboptimal adherence, limited disease knowledge, and poor access to timely clinical support beyond traditional face-to-face delivery [77,78]. Indeed, over the past decade, especially following the COVID-19 outbreak, digital health interventions have become integral to facilitating self-care and continuous disease management in HF patients, particularly when in-person services were limited [79,80]. Digital health interventions encompass telemedicine, mobile health, remote monitoring, big data, and artificial intelligence, which are increasingly used to improve health outcomes [80]. Evidence has shown that telemonitoring interventions are associated with reduced mortality and hospitalization, improved quality of life, and enhanced HF knowledge [81,82]. A systematic review and meta-analysis further indicates that digital interventions significantly improve HF self-care monitoring [83]. Notably, digital health methods may not only improve clinical outcomes but also enhance the psychosocial status of HF patients [84]. For instance, interactive voice response systems enable peer communication to reduce isolation [85]; smartphone-based mobile health platforms integrate cognitive behavioral therapy and mindfulness training, providing personalized guidance to improve anxiety, depression, and self-care behaviors in HF patients [86]. Despite these promising findings, the integration of digital health into HF self-care faces notable challenges and limitations that warrant critical examination. First, the digital divide—disparities in access, connectivity, and literacy—disproportionately affects older adults, who often have cognitive or health-literacy limitations that restrict their engagement with technology-based interventions [87]. This barrier also extends to racial and ethnic minorities, rural residents, and those with lower socioeconomic status, who face lower access to and engagement with digital health technologies, potentially exacerbating rather than mitigating existing disparities [88]. Second, concerns over data privacy, cybersecurity, and the absence of standardized regulatory frameworks impede widespread adoption and erode trust among both patients and clinicians, particularly when sensitive physiological data are transmitted remotely [89]. Third, the extant evidence base remains heterogeneous, with considerable variability in intervention designs, outcome measures, and follow-up durations, which precludes definitive conclusions regarding long-term effectiveness, cost-effectiveness, and optimal implementation strategies. Moreover, most studies have been conducted in highly selective populations, raising questions about generalizability to real-world, diverse settings. Addressing these gaps requires implementation-focused research and patient-centered design to ensure that digital tools benefit all HF patients, not just the technologically privileged.

Looking ahead, continued advances in digital technologies—mobile health, remote monitoring, and artificial intelligence—will extend HF self-care beyond traditional settings, enabling full-cycle management, early warning of deterioration, and personalized interventions. These tools proved valuable during COVID-19 by overcoming follow-up constraints, and integration with big data and next-generation networks promises more proactive, precise care. Yet realizing this potential requires addressing persistent gaps in equity, evidence, and implementation to ensure digital health benefits all HF patients, not just the technologically privileged.

### 4.3. Limitations

This study adopted a bibliometric methodology to explore the research hotspots and emerging frontiers in HF self-care. While this bibliometric analysis is relatively comprehensive and objective, several limitations need to be acknowledged. First, restricting the dataset to English-language articles and reviews indexed in the WoSCC, with further filtering to the categories of “Nursing” or “Cardiac & Cardiovascular Systems,” may have excluded relevant studies from other disciplines, non-English publications, and grey literature, thereby potentially limiting the completeness of the dataset. Second, bibliometric analyses are inherently constrained by database indexing policies and citation-based indicators, which can be influenced by regional citation practices, journal visibility, and temporal factors; self-citation bias and citation lag may further skew metrics, particularly for recent publications. Third, the visualization outputs from VOSviewer and CiteSpace may be influenced by parameter settings (e.g., threshold selection, counting methods, and pruning algorithms); therefore, the findings should be interpreted in light of the parameter choices. Fourth, bibliometric indicators primarily reflect research activity and citation impact rather than clinical approval, guideline endorsement, or real-world adoption. Nevertheless, this approach offers valuable macro-level insights that can inform future translational research.

## 5. Conclusions

This bibliometric analysis characterized the publication landscape and research hotspots of HF self-care research from 1980 to June 2026, based on 2867 WoSCC-indexed publications. A sustained upward trend in publications and citations was observed, accelerating notably after 2008 following the establishment of the situation-specific theory of HF self-care. The USA led in output, impact, and collaboration, with the University of Pennsylvania and Riegel B as the most influential institution and author. The Journal of Cardiovascular Nursing and the European Journal of Cardiovascular Nursing were the primary outlets, underscoring the central role of nursing in this field. Four research hotspots were identified: barriers to self-care and their prognostic impact, psychosocial factors and patient-reported outcomes, intervention strategies and healthcare delivery, and caregiver burden and dyadic interaction. Digital health, particularly telemedicine and mobile health, has emerged as a rapidly evolving frontier. Future efforts should address caregiver burden and dyadic dynamics; advance equitable digital health through patient-centered design and standardized frameworks; and integrate psychosocial screening with tailored management for older adults with multimorbidity and cognitive impairment. These directions may translate research gains into improved clinical outcomes and quality of life for patients with HF.

## Figures and Tables

**Table 1:** Top 10 countries in terms of publications, H-index, and citations.

Rank	Country	Publications	Citations	Average Citations	H-Index
1	USA	1362 (47.51%)	54,297	39.87	104
2	Sweden	239 (8.34%)	11,745	49.14	56
3	Australia	232 (8.09%)	8932	38.50	48
4	Peoples R China	229 (7.99%)	3173	13.86	29
5	United Kingdom	227 (7.92%)	13,619	60.00	59
6	Italy	224 (7.81%)	9859	44.01	55
7	Canada	174 (6.07%)	8973	51.57	49
8	Netherlands	157 (5.48%)	8655	55.13	51
9	Germany	135 (4.71%)	6822	50.53	43
10	South Korea	108 (3.77%)	1879	17.40	24

**Table 2:** Top 10 institutions in terms of publications, H-index, and citations.

Rank	Organization	Publications	Citations	Average Citations	H-Index
1	University of Pennsylvania	177 (6.17%)	11,346	64.10	64
2	University of Kentucky	130 (4.53%)	5761	44.32	43
3	Linköping University	115 (4.01%)	6708	58.33	46
4	University of Rome Tor Vergata	105 (3.66%)	3532	33.64	39
5	Emory University	72 (2.51%)	2980	41.39	32
6	Duke University	67 (2.34%)	3226	48.15	33
7	Johns Hopkins University	67 (2.34%)	2192	32.72	28
8	Australian Catholic University	63 (2.20%)	2531	40.17	30
9	University of California, San Francisco	58 (2.02%)	3687	63.57	33
10	University of North Carolina	57 (1.99%)	4246	74.49	29

**Table 3:** Top 10 authors in terms of publications, H-index, and citations.

Rank	Author	Publications	Citations	H-Index
1	Riegel, B	135	10,730	63
2	Vellone, E	102	3738	34
3	Moser, DK	100	5379	43
4	Jaarsma, T	75	5063	44
5	Lee, CS	70	4868	35
6	Lennie, TA	60	3026	32
7	Alvaro, R	52	2178	28
8	Stromberg, A	50	3412	34
9	Davidson, PM	40	892	20
10	Dickson, VV	38	3330	26

**Table 4:** Top 10 journals in terms of publications, JCR.

Rank	Journal	Publications	JCR
1	Journal of Cardiovascular Nursing	308	Q2
2	European Journal of Cardiovascular Nursing	227	Q1
3	Heart & Lung	170	Q2
4	Journal of Clinical Nursing	90	Q1
5	European Journal of Heart Failure	83	Q1
6	Journal of Cardiac Failure	83	Q1
7	Journal of Advanced Nursing	67	Q1
8	International Journal of Nursing Studies	66	Q1
9	BMC Cardiovascular Disorders	60	Q2
10	ESC Heart Failure	55	Q2

**Table 5:** Top 10 co-cited journals in terms of total citations.

Rank	Journal	Citations	Total Link Strength	JCR
1	Journal of Cardiovascular Nursing	5040	59,867	Q2
2	Circulation	4921	80,499	Q1
3	European Journal of Heart Failure	4022	64,875	Q1
4	Journal of the American College of Cardiology	3554	68,569	Q1
5	Journal of Cardiac Failure	3081	50,991	Q1
6	Heart & Lung	3042	42,029	Q2
7	European Journal of Cardiovascular Nursing	2690	34,307	Q1
8	European Heart Journal	2606	49,410	Q1
9	New England Journal of Medicine	1769	45,843	Q1
10	JAMA	1754	31,960	Q1

**Table 6:** Top 20 keywords in HF self-care.

Rank	Keyword	Occurrences	Total Link Strength
1	heart failure	1862	12,037
2	self-care	1104	7889
3	quality of life	754	5596
4	management	623	4298
5	outcomes	432	3092
6	depression	354	2767
7	intervention	347	2721
8	mortality	330	2314
9	association	316	2403
10	care	291	1904
11	disease	274	2007
12	self-management	270	1875
13	adherence	248	1820
14	older adults	243	1915
15	health	221	1643
16	knowledge	220	1666
17	risk	211	1399
18	adults	206	1622
19	impact	205	1417
20	symptoms	189	1371

**Table 7:** Top 10 funding agencies for HF self-care research.

Rank	Funding Agencies	Publications
1	United States Department of Health & Human Services	475
2	National Institutes of Health (NIH), USA	464
3	NIH National Heart, Lung, and Blood Institute (NHLBI)	182
4	NIH National Institute of Nursing Research (NINR)	181
5	American Heart Association	77
6	NIH National Center for Advancing Translational Sciences (NCATS)	70
7	NIH National Institute on Aging (NIA)	56
8	National Research Foundation of Korea	46
9	Japan Society for the Promotion of Science	39
10	Ministry of Education, Culture, Sports, Science and Technology (MEXT), Japan	39

## Data Availability

No new data were created or analyzed in this study. Data sharing is not applicable to this article.

## Supplementary Materials

The following supporting information can be downloaded at: https://www.mdpi.com/article/10.3390/healthcare14172881/s1, Supplementary Materials: Thesaurus File.

## Abbreviations

The following abbreviations are used in this manuscript:

HF	Heart Failure
ESC	European Society of Cardiology
WoSCC	Web of Science Core Collection
USA	The United States
JCR	Journal Citation Reports
