# Supplementary material for: Global Trends in Heart Failure Self-Care Research: A Bibliometric and Visual Analysis
Source: Healthcare (Basel). 2026 Sep 7;14(17):2881. doi: 10.3390/healthcare14172881 (PMC13565471; doi:10.3390/healthcare14172881)
Supplement: Supplementary file 1 [file healthcare-14-02881-s001.zip › healthcare-4364148-supplementary.pdf]

Thesaurus for VOSviewer Analysis

Geographical Locations

label	replace by
england	united kingdom
scotland	united kingdom
wales	united kingdom
north ireland	united kingdom
turkiye	turkey

Affiliations / Institutions

label	replace by
univ north carolina	univ n carolina
gothenburg univ	univ gothenburg
umea univ	univ umea
western sydney univ	univ western sydney
st vincents hosp	st vincent hosp

Keywords

atrial-fibrillation	atrial fibrillation
behaviors	behavior
cardiovascular-disease	cardiovascular disease
caregivers	caregiver
chronic heart-failure	chronic heart failure
elderly	older adults
heart-failure	heart failure
health-status	health status
interventions	intervention
metaanalysis	meta-analysis
mhealth	mobile health
myocardial-infarction	myocardial infarction
older-adults	older adults
older patients	older adults
physical-activity	physical activity
quality-of-life	quality of life
randomized controlled-trial	randomized controlled trial
randomized-trial	randomized controlled trial
rehospitalization	readmission
self care	self-care

Thesaurus for Citespace Analysis

replace by	label
heart failure	heart-failure
of life	of-life
self-care	self care
self-care behaviors	self care behaviors

outcome	outcm
clinical outcome	clinical outcm
clinical outcome	clinical-outcm
depression	depressive symptoms
intervention	interventions
elderly patients	elderly-patients
older adults	older-adults
elderly patients	older patients
readmission	readmissions
hospital readmission	hospital readmissions
randomized controlled trial	randomized controlled-trial
risk factors	risk-factors
cardiovascular disease	cardiovascular diseases
chronic disease	chronic illness
obstructive pulmonary disease	chronic obstructive pulmonary disease
guidelines	guideline
behaviors	behavior
myocardial infarction	myocardial-infarction
self efficacy	self efficacy
perception	perceptions
converting enzyme inhibitors	ace-inhibitors
person centered care	person-centred care
